# Effects of Premature Ventricular Complex Burden on Left Ventricular Global Longitudinal Strain in Patients without Structural Heart Disease

**DOI:** 10.3390/jcm13061796

**Published:** 2024-03-20

**Authors:** Arslan Sukru, Arabaci H. Ozan, Deniz M. Furkan, Gokce M. Emin, Arslan Seyma, Oktay Veysel, Yıldız Mustafa, Uzunhasan Isıl

**Affiliations:** 1 Department of Cardiology, Cardiology Institute, Istanbul University-Cerrahpasa, 34303 Istanbul, Turkey; sukru.arslan@iuc.edu.tr (A.S.); drfurkandeniz@gmail.com (D.M.F.); mehmet_emin_gkc@hotmail.com (G.M.E.); drvoktay@gmail.com (O.V.); mustafayilldiz@yahoo.com (Y.M.); isil.uzunhasan@gmail.com (U.I.); 2 Republic of Turkey Ministry of Health, 06800 Ankara, Turkey; sheyma87@gmail.com

**Keywords:** idiopathic PVC, global longitudinal strain, PVC burden

## Abstract

**Background:** Evaluation of left ventricular (LV) function in patients with idiopathic premature ventricular contraction (PVC) with preserved LV ejection fraction (LVEF), especially in the subclinical stage, may be of great importance in terms of directing early treatment. **Methods:** A total of 122 patients, retrospectively recruited, were divided into three groups according to PVC burden: Group 1; 5% ≤ PVC < 10%, Group 2; 10% ≤ PVC < 15%, and Group 3; 15% ≤ PVC. Transthoracic echocardiography (TTE) was performed to evaluate LV parameters. **Results:** LV-GLS (Global longitudinal strain) was found to be significantly lower in groups 2 and 3 with high PVC burden (18.9% ± 1.4, 17.5% ± 2.1, 16.3% ± 1.3; *p* < 0.001, respectively). Correlation analysis revealed a positive and significant correlation between PVC burden and deterioration in LV-GLS (r: 0.536; *p* < 0.001). In addition, PVC burden was found to be an independent predictor of LV-GLS deterioration in multiple linear regression analysis (β: 0.525, *p* < 0.001). The ROC curve analysis demonstrated that a PVC burden cut-off value of 8.4% was associated with a LV-GLS deterioration greater than −18, with a specificity of 75.4% and a sensitivity of 75.4% (AUC: 0.81 [0.73–0.88] 95% CI; *p* < 0.001). **Conclusions:** PVC burden was an independent predictor of deterioration in LV-GLS. The presence of LV-GLS deterioration due to PVC burden emphasizes the necessity for closer patient monitoring, observation of the response to pharmacological treatment, and evaluation of early invasive treatment strategies in selected patient groups.

## 1. Introduction

Idiopathic ventricular arrhythmias are defined as rhythm disorders detected in individuals without structural heart disease or genetic ion channel defects. Idiopathic premature ventricular contractions (PVCs) are rhythm abnormalities that are commonly encountered in clinical practice. They often present asymptomatically, whereas they might rarely lead to syncope or sudden cardiac death [1]. Idiopathic PVCs are generally considered benign; however, studies have shown that increasing PVC burden can lead to impairment in left ventricular function [2,3,4,5]. The PVC burden, QRS duration, short coupling interval duration, presence (and, if present, frequency) of non-sustained ventricular tachycardia, and presence of PVCs with different morphologies have been reported to predict left ventricular dysfunction [6,7,8]. It has been demonstrated in the literature that increasing PVC frequency can lead to ventricular dilatation and reduced ejection fraction (EF) [4,5]. In patients with preserved LVEF, data concerning the association between premature PVC burden and functional impairment in the left ventricle are limited. Recently, the use of speckle tracking echocardiography has been associated with the detection of subclinical ventricular dysfunction by assessing left ventricular global longitudinal strain (LV-GLS). However, the cut-off values for PVC burden that cause impairment in left ventricular function have not yet been clearly defined [8,9,10]. In our study, we aimed to investigate the effects of PVC burden on LV-GLS and myocardial performance index (MPI) in patients with normal LVEF diagnosed with idiopathic PVCs. Additionally, we aimed to determine the cut-off values of PVC burden that predict impairment in LV-GLS.

## 2. Materials and Methods

### 2.1. Study Population

Our study included consecutive patients who were presented to our arrhythmia clinic between 2019 and 2022 with a diagnosis of idiopathic frequent PVCs. Analysis was conducted on a cohort comprising 147 patients with an idiopathic PVC burden exceeding 5% based on a 24 h rhythm Holter recording.

**Figure 1:**
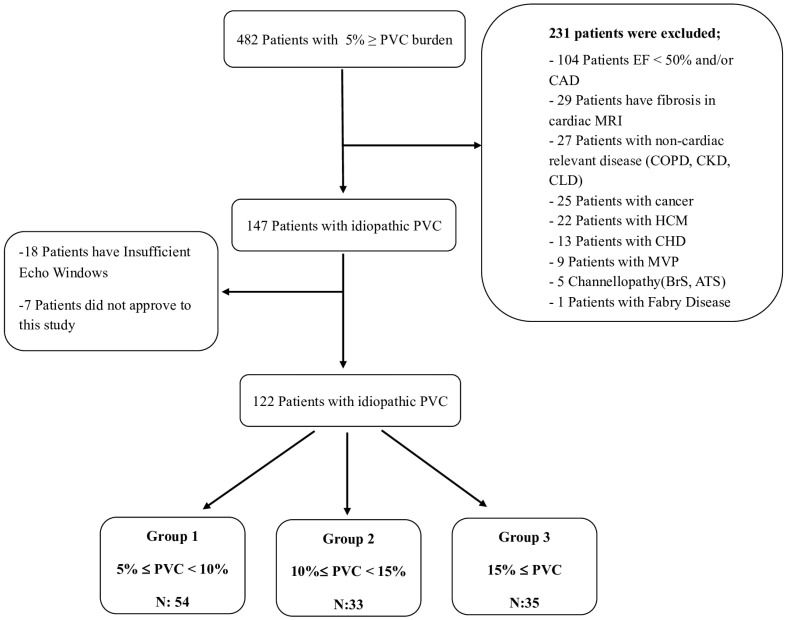
Trial flow chart. ATS: Andersen–Tawil Syndrome, BrS: Brugada Syndrome, CAD: Coronary artery disease, CHD: Congenital heart disease, CKD: Chronic kidney disease, CLD: Chronic lung disease, COPD: Chronic obstructive pulmonary disease, Echo: Echocardiography, EF: Ejection fraction, HCM: Hypertrophic cardiomyopathy, MVP: Mitral valve prolapse, PVC: Premature ventricular contractions.

The inclusion criteria of our study were defined as follows: being older than 18 years of age, having a PVC burden of over 5%, and providing informed voluntary consent. Exclusion criteria were patients with a diagnosis of coronary artery disease (CAD), cardiomyopathy, moderate to severe valvular heart disease (VHD), congenital heart disease (CHD), diagnosed arrhythmic genetic syndromes, ion channel defects, left ventricular hypertrophy, mitral valve prolapse (MVP), left ventricular ejection fraction (LVEF) < 50%, presence of fibrosis on cardiac MRI, non-cardiac relevant disease (chronic obstructive pulmonary disease, chronic kidney disease, chronic liver disease), or cancer diagnosis. These patients were excluded from the study. The definition of idiopathic frequent PVC was determined to be PVCs outside the exclusion criteria stated above. Informed consent was obtained from each patient, and the study was conducted according to the guidelines of the Declaration of Helsinki 1975 and approved by the Institutional Review Board of Istanbul University-Cerrahpasa (E-69291215-900-16420 and 2 March 2021). A total of 122 patients included in the study were divided into three groups based on the PVC burden. A flow chart of this study is given in Figure 1. Patients were classified as symptomatic or asymptomatic based on the presence of symptoms. Symptomatic patients were further divided into those with typical complaints of palpitations and those with atypical symptoms. Atypical symptoms included dyspnea, fatigue, chest pain, dizziness, and fainting [11]. The demographic characteristics of the patients were obtained by reviewing the hospital automation system notes, calling patients for follow-up visits, and conducting face-to-face interviews, as well as scanning patient medical records.

### 2.2. Electrocardiography (ECG) and Rhythm Holter Analysis

The baseline heart rates, QTc intervals, and presence or absence of PVCs were evaluated in the available 12-lead ECG recordings of the patients. The QTc intervals were calculated using the Bazett formula (QTc = QT/√R − R).

In the 24 h rhythm Holter recordings of the patients reviewed and included in the study, along with PVC burden, the average heart rate, minimum heart rate, maximum heart rate, presence of non-sustained ventricular tachycardia (NSVT), and its morphology were noted. The PVC burden was obtained by dividing the number of PVCs detected in the 24 h rhythm Holter recordings by the total QRS count calculated within 24 h, and it was recorded in the patient files.

The included patients were divided into three groups based on their PVC burden for evaluation. The groups were defined as follows based on PVC burden: Group 1 included patients with 5% ≤ PVC < 10%, Group 2 included patients with 10% ≤ PVC < 15%, and Group 3 included patients with PVC burden ≥15%. In the classification of patients into three different groups on the basis of PVC burden, the designated PVC burden percentages were determined by evaluation in accordance with ESC guidelines [11]. In these guidelines, the impact of a PVC burden in excess of 5–10% on cardiac outcomes was discussed, so these PVC burden values were used to stratify patients [11].

### 2.3. Transthoracic Echocardiographic (TTE) Evaluation

The echocardiographic measurements and evaluations were performed by two experienced cardiologists who were blinded to patient characteristics. Transthoracic echocardiography (TTE) recordings of the patients were obtained using the Philips Epiq 7 (Philips Medical Systems, Andover, MA, USA) device. In TTE, each patient’s parasternal long-axis and short-axis views, as well as apical two-chamber, apical three-chamber, and apical four-chamber views, were obtained under ECG guidance. LVEF was calculated by evaluating three consecutive beats without PVC and using the modified Simpson’s method. Images were obtained using the techniques recommended by the European Association of Echocardiography (EAE)/American Society of Echocardiography (ASE) guidelines [12]. Apical two-chamber, apical three-chamber, and apical four-chamber recordings were analyzed for LV-GLS. LV-GLS values were calculated and recorded using the QLAB-CMQ Autostrain computer program. For the calculation of the MPI, measurements of the mitral valve inflow velocities were obtained using a pulse wave Doppler cursor positioned below the plane of the aortic valve in the apical five-chamber view. All measurements were recorded as the average of measurements obtained over three consecutive cardiac cycles.

The isovolumetric relaxation time (IVRT) was measured as the time interval from aortic valve closure to mitral valve opening. The isovolumetric contraction time (IVCT) was measured as the time interval from mitral valve closure to aortic valve opening. The ejection time (ET) was measured as the duration from the opening to the closure of the aortic valve in the left ventricular outflow velocity profile. The myocardial performance index (MPI) was calculated by dividing the sum of IVRT and IVCT by ET. The obtained MPI values and GLS values were recorded in the patient files.

The study protocol was approved by the local ethics committee. Informed consent was obtained from each patient.

### 2.4. Statistical Analysis

The data obtained in the study were analyzed using SPSS v.25 software (SPSS Inc., Chicago, IL, USA). A normal distribution analysis of the data was performed using the Shapiro–Wilk (W) test. Continuous variables that showed a normal distribution were presented as mean ± standard deviation, while non-normally distributed variables were reported as median (minimum-maximum) values. Categorical variables were presented in tables as frequencies and percentages. The normally distributed continuous variables among the three groups in our study were evaluated using the one-way ANOVA test, while the non-normally distributed data were evaluated using the Kruskal–Wallis H test.

Categorical data were compared using the chi-square test. The relationship between the number of PVCs, PVC burden, LV-GLS, and MPI index parameters in our study was evaluated using correlation analysis. Since three of these parameters did not follow a normal distribution, correlation coefficients, and statistical significance were calculated using the Spearman test. The different cut-off values for PVC count and PVC burden predicting deterioration in GLS were examined using Receiver Operating Characteristics (ROCs) curve analysis. In our study, the LV-GLS cut-off values, which were taken as levels and evaluated in the ROC curve analysis, were determined as −17%, −18%, −19%, and −20%. These values were established by obtaining data from studies identifying specific cut-off values associated with impairment in LV-GLS levels in the literature [10,13,14]. Specificity and sensitivity calculations were performed in the presence of a significant threshold value. A multiple linear regression model was constructed to identify the parameters predicting the decrease in LV-GLS. With this model, the determination of independent variables affecting the dependent variable was achieved in LV-GLS. A total of 20 patients were randomly selected for intra- and interobserver variability analysis for GLS values, according to this; the Bland–Altman analysis was performed (Figure S1). Statistical significance was considered at *p* < 0.05 in all analyses.

## 3. Results

### 3.1. Clinical and Demographical Features and Laboratory Parameters:

A total of 147 patients with an idiopathic PVC burden of more than 5% were analyzed. Among them, 122 patients who met the inclusion criteria, had appropriate echocardiographic images, and had no familial relationship with each other were selected and included in the study. Approximately 57% of the patients included in our study were female, and the mean age of the entire population was 45.7 ± 12.7 years. Furthermore, 69.7% of the included patients had palpitation complaints (Table 1), The evaluation of the groups in terms of baseline clinical and demographic parameters is presented in Table 1. A significant difference in body mass index (BMI) was observed among the groups, with the difference originating from the higher BMI in Group 2 (Table 1). In the study, it was observed that the average duration of symptoms in patients was 15 months; however, no significant difference was detected among the groups (Table 1). Approximately 62% of the patients included in the study were evaluated for the presence of structural heart diseases and cardiac fibrosis using cardiac MRI, and none of the patients exhibited these findings. Furthermore, myocardial perfusion scintigraphy (MPS) was performed on a total of 34 patients across the three groups, and no pathological findings indicative of ischemia were detected. No significant difference was found among the groups regarding other clinical and demographic parameters. No significant difference was observed among the groups in terms of laboratory parameters (Table S1).

The evaluation of the groups in terms of electrocardiographic (ECG) and echocardiographic (Echo) parameters in our study is presented in Table 2. The presence of PVCs in the baseline ECG recordings were more frequently observed in patients in Group 2 and Group 3 with a PVC burden of 10% or higher (Table 2). In patients grouped according to PVC burden, the mean PVC burden was determined to be 6.2% for Group 1, 11.3% for Group 2, and 22.5% for Group 3 (*p* < 0.001, Table 2). Similarly, on the 24 h ambulatory rhythm Holter recordings, NSVT episodes were more frequently observed in patients in Group 2 and Group 3, showing statistically significant differences (*p* = 0.006). There was no significant difference among the groups in terms of the rate of polymorphic PVCs in the Holter recordings, QTc values in the baseline ECG recordings, and other ECG and Holter parameters. The right ventricular end-diastolic diameter (RVd) was significantly lower in Group 2 patients compared with the other groups (*p* = 0.044, Table 2). The myocardial performance index was not significantly different across the three groups (Table 2). When comparing LV-GLS values among the groups, a statistically significant difference was observed (−18.9% ± 1.4, −17.5% ± 2.1, −16.3% ± 1.3, *p* < 0.001, respectively). No significant difference was found among the groups in terms of other Echo parameters.

### 3.2. Correlation and Multiple Linear Regression Analysis

The correlation analyses between PVC burden, PVC count, LV-GLS, and MPI values are presented in Table 3. An increase in PVC count and PVC burden showed a positive, moderate-level, and significant correlation with deterioration in GLS values (r: 0.555; *p* < 0.001, r: 0.536; *p* < 0.001, respectively). Similarly, an increase in PVC count and PVC burden showed a negative, weak-level, and significant correlation with MPI values (r: 0.220; *p*: 0.015, r: 0.219; *p*: 0.015, respectively). In addition, a correlation analysis was conducted to assess the relationship between the duration of symptoms in patients and LV-GLS values. Despite being weak, a positive correlation was identified between symptom duration and LV-GLS (r: 0.195; *p*: 0.032).

According to the multiple linear regression modeling, ten parameters (PVC (%), hemoglobin, ECG QTc, left atrium (PLAX), left ventricular end-diastolic diameter, MPI, body mass index, age, tricuspid annular plane systolic excursion, RV end-diastolic diameter) were assessed for their ability to predict the decrease in LV-GLS. It was determined that the percentage (%) of PVC independently predicted the decrease in LV-GLS (β: 0.525, *p* < 0.001; adjusted R^2^: 0.340) (Table 4). Other parameters were not found to be statistically significant in this modeling.

### 3.3. ROC Analysis

**Figure 2:**
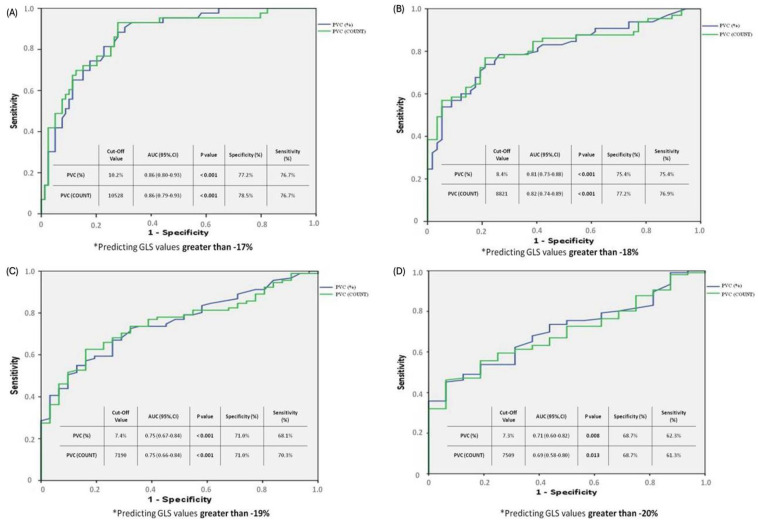
The ROC curve analysis modelling examples conducted to determine the cut-off values of PVC burden and PVC count predicting deterioration in LV-GLS. (**A**–**D**); GLS; global longitudinal strain, PVC: premature ventricular contraction. (**A**) * It demonstrates the PVC burden (%) and PVC count predicting the deterioration at −17% and greater in the GLS value. According to ROC analysis, a PVC burden of 10.2% or higher can predict a decrease in GLS value greater than −17% with a specificity of 77.2% and a sensitivity of 77.6%. (**B**) * It demonstrates the PVC burden (%) and PVC count predicting the deterioration at −18% and greater in the GLS value. According to ROC analysis, a PVC burden of 8.4% or higher can predict a decrease in GLS value greater than −18% with a specificity of 75.4% and a sensitivity of 75.4%. (**C**) * It demonstrates the PVC burden (%) and PVC count predicting the deterioration at −19% and greater in the GLS value. According to ROC analysis, a PVC burden of 7.4% or higher can predict a decrease in GLS value greater than −19% with a specificity of 71.0% and a sensitivity of 68.1%. (**D**) * It demonstrates the PVC burden (%) and PVC count predicting the deterioration at −20% and greater in the GLS value. According to ROC analysis, a PVC burden of 7.3% or higher can predict a decrease in GLS value greater than −20% with a specificity of 68.7% and a sensitivity of 62.3%.

The results of the ROC curve analysis modeling examples conducted to determine the cut-off values of PVC burden and PVC count predicting deterioration in LV-GLS, based on different LV-GLS values (−17, −18, −19, −20), are shown in Figure 2. In the first analysis (Figure 2A), a PVC burden cut-off value of 10.2% was found to be associated with a LV-GLS deterioration greater than −17, with a specificity of 77.2% and a sensitivity of 77.6% (PVC 10.2% cut-off value, AUC: 0.86 [0.80–0.93] 95% CI; *p* < 0.001). In the second analysis (Figure 2B), a PVC burden cut-off value of 8.4% was associated with an LV-GLS deterioration greater than −18, with a specificity of 75.4% and a sensitivity of 75.4% (PVC 8.4% cut-off value, AUC: 0.81 [0.73–0.88] 95% CI; *p* < 0.001). In the third analysis (Figure 2C), a PVC burden cut-off value of 7.4% was associated with an LV-GLS deterioration greater than −19, with a specificity of 71% and a sensitivity of 68.1% (PVC 7.4% cut-off value, AUC: 0.75 [0.67–0.84] 95% CI; *p* < 0.001). Finally, in the fourth analysis (Figure 2D), a PVC burden cut-off value of 7.3% predicted a GLS deterioration greater than −20, with a specificity of 68.7% and a sensitivity of 62.3% (PVC 7.3% cut-off value, AUC: 0.71 [0.60–0.82] 95% CI; *p* < 0.008).

## 4. Discussion

In our study, a significant difference was found in the LV-GLS values between the three groups determined according to the PVC burden. The correlation analyses revealed a moderate-level significant correlation between the PVC burden and the deterioration of LV-GLS values. In the ROC analysis models conducted to determine the cut-off values of PVC burden predicting the deterioration of LV-GLS values, it was revealed that a PVC burden above 8.4% particularly predicted a decrease of more than −18 in LV-GLS values with a specificity and sensitivity rate of 75.4%. Furthermore, we determined that aPVC burden exceeding 10.2% was revealed to be associated with a deterioration of more than −17 in LV-GLS values with a specificity of 77.2% and sensitivity of 77.6%. Similarly, in correlation analyses, a weak-level significant negative correlation was observed between the PVC burden and the MPI values. In the multiple linear regression model, PVC burden was identified as an independent predictor for the reduction in LV-GLS.

Upon reviewing the literature, Ling Y. et al.’s study compared 38 patients with a mean PVC burden of 22.8% ± 9 and 39 healthy control subjects with normal LV-EF values, revealing a significant decrease in strain values compared to the control group [15]. In the study conducted by Ban JE. et al., 127 patients were analyzed without structural heart disease who had a daily PVC burden of more than 10%. They reported LVEF < 50% in 22% of the patients and stated that a PVC burden above 26% predicted impaired LV function with 71% specificity and 79% sensitivity [16]. In this study, a PVC burden of 10% or higher has been associated with a decrease in LVEF, indicating a clinically significant major LV impairment. Indeed, this impairment is correlated with the PVC burden; in our study, a PVC burden of 5% or higher is associated with preserved LVEF but leads to a significant subclinical deterioration in LV-GLS. As the PVC burden increases, the negative impact on LV functions becomes evident. A similar study was conducted by Baman TS et al., where they included 174 patients and reported a mean LVEF of 37% ± 10. They indicated that 33% of the study population consisted of patients with reduced LVEF and a PVC burden above 24%, which predicted impaired left ventricular function with 78% specificity and 79% sensitivity. Furthermore, in this study, they reported that the lowest PVC burden leading to reversible cardiomyopathy was above 10% [17]. In our study, particularly in patients in Group 3, the mean PVC burden was calculated as 22.5%, with only one patient exhibiting a notably high PVC burden of 39.8%. Despite this elevated PVC burden, the absence of a decrease in LVEF or LV end-diastolic diameter is surprising. The relatively shorter duration of symptoms in our patients, the absence of scar on CMR, and the optimal use of medical treatments may have resulted in a decrease in LV-GLS indicating subclinical damage only, rather than overt LV dysfunction. In a study conducted by Lie ØH et al., involving 52 patients with outflow tract PVCs, they reported that a PVC burden above 8% could be associated with impaired LV-GLS, defined as values greater than −18% [14]. Additionally, Shanmugam N. et al. demonstrated in their published cases that even a PVC burden above 4% could lead to impairments in left ventricular function [18].

Recently, strain echocardiography, which enables the detection of subclinical impairments without a decline in LV-EF, has become widely used. Although some studies have provided cut-off values for LV-GLS values [14], there is no specific LV-GLS cut-off value indicating impairment for our patient group, as it has not been studied.

The association between the PVC burden and the decrease in LV-GLS has been emphasized in numerous studies [10,14,15,17]. Upon examining parameters predicting this decrease, clinical parameters such as age and BMI, as well as various electrocardiogram parameters and laboratory findings, were found to be non-significant. In accordance with our conducted multiple linear regression modeling, we identified that the only independent parameter predicting the decrease in LV-GLS is the PVC burden.

Another parameter reflecting the left ventricular function is the MPI value. Studies on heart failure, acute coronary syndrome, hypertension, and diabetic patients have reported that a decrease in MPI values is associated with the severity of coronary artery disease, mortality, and morbidity [19,20,21]. The effects of PVC burden on MPI in patients with preserved LV-EF have not been extensively studied. In our study, no significant difference was found among groups in terms of MPI, when examined in distinct, to some extent arbitrary, PVC frequency categories. However, correlation analyses revealed a weak but significant correlation between PVC burden, frequency, and MPI values, indicating impairment.

The duration of symptoms related to PVCs can influence LV-GLS values, as much as the frequency of PVCs. In a previous study, a symptom duration of 60 months or more was identified as a predictor for PVC-induced cardiomyopathy in patients with idiopathic PVCs [22]**.** In our study, we also detected a weak but discernible correlation between the duration of symptoms related to PVCs and LV-GLS values.

Highlighting the strengths of our study, it possesses a reasonable number of patients compared to strain echocardiography studies, albeit fewer, and focuses on the relationship between PVC burden and LV-GLS. Deterioration in LV-GLS and other outcome analyses were not solely classified based on PVC burden but were also supported by correlation analyses. The cut-off value predicting LV-GLS deterioration of more than −18% due to PVC burden was determined through ROC curve analysis with high sensitivity and specificity. Finally, the consistency of the measured LV-GLS values between operators in our study was determined using the Bland–Altman Analysis.

### Limitations of the Study

The study is constrained by several limitations. It maintains a single-center design, records Holter data from only three leads, and measures the burden of PVC by not relying on rhythm Holter recording for more than 24 h. Moreover, the definitive determination of PVC origin is hindered by a suboptimal PVC detection rate on ECGs, registering below 35%. Also, a significant majority of enrolled patients declined participation in the electrophysiological study (EPS), thus impeding comprehensive insights into PVC origins through invasive means. Furthermore, notwithstanding the accessibility of symptom duration data for patients, this study is subject to other limitations, including the absence of long-term follow-up and an evaluation of the influence of LV-GLS values on major cardiovascular endpoints.

## 5. Conclusions

In our study, significant impairment was observed in LV-GLS values in groups with a high PVC burden. Increased PVC burden and frequency were correlated with impaired LV-GLS values; additionally, an increase in PVC burden was identified as an independent predictor of deterioration in LV-GLS. Furthermore, contrary to previous literature relying on LVEF, it was found that even lower levels of PVC burden could be associated with impairments in left ventricular function, measured as GLS, in individuals with preserved LVEF and structurally normal hearts. This suggests that, in this cohort of patients, early consideration of closer monitoring and evaluation of pharmacotherapy or even invasive procedures such as PVC focus ablation may be considered. However, the clarity of invasive treatments based solely on the significant deterioration in LV-GLS in patients with preserved LVEF remains uncertain, necessitating the need for more current data on this matter. Finally, in monitoring this patient group, strain echocardiography should be prioritized over standard echocardiography.

## Figures and Tables

**Table 1:** Baseline clinical and demographic characteristics of patients according to groups.

	Group 1 N: 54	Group 2 N: 33	Group 3 N: 35	*p* Value
Age (years)	46.83 ± 12.6	46.94 ± 8.8	42.83 ± 14.8	0.280
Male, n (%)	20 (37.0%)	16 (48.5%)	17 (48.6%)	0.445
Hypertension, n (%)	23 (42.6%)	10 (30.3%)	11 (31.4%)	0.407
Diabetes Mellitus, n (%)	12 (22.2%)	5 (15.2%)	4 (11.4%)	0.392
Hyperlipidemia, n (%)	13 (24.1%)	4 (12.1%)	8 (22.9%)	0.374
Previous CVA/TIA, n (%)	2 (3.7%)	0	1 (2.9%)	0.548
Current smoking, n (%)	28 (51.9%)	23 (69.7%)	20 (57.1%)	0.259
Sudden death in family, n (%)	10 (18.5%)	5 (15.2%)	12 (34.3%)	0.114
Drug Usage, n (%)				
B-Blockers	35 (64.8%)	23 (69.7%)	30 (85.7%)	0.093
CCB	9 (16.7%)	5 (15.2%)	11 (31.4%)	0.163
Class Ic AAD	9 (16.7%)	5 (15.2%)	12 (34.3%)	0.084
Class III AAD	4 (7.4%)	2 (6.1%)	4 (11.4%)	0.694
BMI	25.4 ± 2.7	26.6 ± 3.5	24.7 ± 2.7	0.029
Symptoms				
Asymptomatic, n (%)	4 (7.4%)	2 (6.3%)	2 (5.7%)	0.181
Palpitations, n (%)	39 (72.2%)	26 (81.3%)	20 (57.1%)	
Atypical symptoms, n (%)	11 (20.4%)	4 (12.5%)	13 (37.1%)	
Symptoms duration, month	15.57 ± 8.2	14.94 ± 6.8	18.54 ± 9.7	0.123
CAG, n (%)	12 (22.2%)	7 (21.2%)	13 (37.1%)	0.220
MPS, n (%)	12 (22.2%)	8 (24.2%)	14 (40.0%)	0.162

median (min-max). AAD: Antiarrhythmic drug, BMI: Body mass index, CAG: Coronary angiography, CCB: Calcium channel blockers, CVA: Cerebrovascular accident, MPS: Myocardial perfusion scintigraphy, PVC: Premature ventricular complex, TIA: Transient ischemic attack.

**Table 2:** Baseline electrocardiographic and echocardiographic parameters of patients in the study groups.

	Group 1 N: 54	Group 2 N: 33	Group 3 N: 35	*p* Value
PVC Count/day	5890 ± 1064	10778 ± 2069	23689 ± 6452	<0.001
PVC Burden (%) *	6.2% (5.1–8.9)	11.3% (10–14.8)	22.5% (16.1–39.8)	<0.001
ECG-PVC, n (%)	12 (22.2%)	14 (42.4%)	18 (51.4%)	0.013
QTc (msc)	419.7 ±40.1	425.1 ± 32.8	422.4 ± 30.3	0.789
BBB, n (%)	10(18.5%)	10(30.3%)	10(28,6%)	0.377
Min. HR/min	51.3 ± 14.6	49.5 ± 5.9	48.9 ± 6.6	0.490
Max. HR/min	128.7 ± 21.3	130.6 ± 15.1	132.2 ± 18.1	0.781
Average HR/min	74.6 ± 8.2	75.8 ± 9.5	76.3 ± 7.2	0.598
Polymorphic PVC, n (%)	5 (23.8%)	2 (20.0%)	1 (7.1%)	0.440
NSVT, n (%)	8(14.8%)	13(39.4%)	15(42.9%)	0.006
LA, (mm)	36.6 ± 4.6	37.4 ± 5.8	38.9 ± 4.8	0.098
LVd, (mm)	48.0 ± 3.3	50.5 ± 4.9	49.9 ± 7.7	0.066
RVd, (mm)	22.3 ± 1.9	23.5 ± 1.9	22.6 ± 2.1	0.044
IVS, (mm)	9.6 ± 1.2	9.7 ± 1.4	9.7 ± 1.1	0.874
TAPSE, (cm)	2.3 ± 0.3	2.2 ± 0.3	2.2 ± 0.2	0.737
sPAP (mmHg)	23.9 ± 4.4	23.8 ± 4.3	24.6 ± 4.3	0.625
MPI Index	0.50 ± 0.06	0.48 ± 0.08	0.47 ± 0.06	0.143
LV-GLS %	−18.9 ± 1.4	−17.5 ± 2.1	−16.3 ± 1.3	<0.001

* median (min-max). BBB: bundle branch block, ECG: electrocardiogram, HR; heart rate, IVS: interventricular septum, LA: left atrium, LVd: left ventricular end-diastolic diameter, LV-GLS: left ventricular global longitudinal strain, MPI: myocardial performance index, NSVT: non-sustained ventricular tachycardia, PVC: premature ventricular complex, QTc: corrected QT interval, RVd: right ventricular diameter, sPAP: systolic pulmonary arterial pressure, TAPSE: tricuspid annular plane systolic excursion.

**Table 3:** Results of correlation analysis (PVC percentages and counts with LV-GLS and MPI Levels. Symptom duration with LV-GLS).

	N	Correlation Coefficient (r)	*p* Value
PVC (%) and LV-GLS	122	0.536 *	<0.001
PVC counts and LV-GLS	122	0.555 *	<0.001
PVC (%) and MPI	122	−0.219 **	0.015
PVC counts and MPI	122	−0.220 **	0.015
Symptom duration and LV-GLS	122	0.195 **	0.032

* Moderately Correlated. ** Weakly Correlated. LV-GLS: left ventricular global longitudinal strain, MPI: myocardial performance index, PVC: premature ventricular complex.

**Table 4:** The multiple linear regression analysis.

	B	Std. Error_B_	Beta	t	Sig. (*p*)	Lower Bound (Min)	Upper Bound (Max)	VIF
Predictors								
PVC (%)	0.121	0.018	0.525	6.540	<0.001	0.084	0.157	1.194
Hb	−0.050	0.103	−0.038	−0.486	0.628	−0.253	0.153	1.149
ECG QTc	0.005	0.004	0.091	1.204	0.231	−0.003	0.013	1.052
LA	0.058	0.043	0.152	1.357	0.177	−0.027	0.143	2.314
LVd	0.012	0.036	0.033	0.737	0.737	−0.060	0.084	1.744
MPI	−0.539	2.273	−0.019	−0.237	0.813	−5.044	3.965	1.137
BMI	0.011	0.062	0.016	0.171	0.864	−0.113	0.134	1.651
AGE	0.021	0.015	0.133	1.371	0.173	−0.009	0.051	1.747
TAPSE	−0.511	0.546	−0.073	−0.936	0.351	−1.592	0.571	1.122
RVd	0.067	0.081	0.070	0.835	0.406	−0.092	0.227	1.289
R^2^ = 0.400 Adjusted R^2^ = 0.346								

BMI: body mass index, ECG: electrocardiogram, Hb: hemoglobin, LA: left atrium, LVd: left ventricular diastolic diameter, max: maximum, min: minimum, MPI: myocardial performance index, PVC: premature ventricular complex, QTc: corrected QT interval, RVd: right ventricular diastolic diameter, TAPSE: tricuspid annular plane systolic excursion.

## Data Availability

The original contributions presented in the study are included in the article/Supplementary Material, further inquiries can be directed to the corresponding author.

## Supplementary Materials

The following supporting information can be downloaded at: https://www.mdpi.com/article/10.3390/jcm13061796/s1, Figure S1: The Blant–Altman Analysis. Table S1: Baseline Biochemical Characteristics of Patients in The Groups.
