# Supplementary material for: Effects of Premature Ventricular Complex Burden on Left Ventricular Global Longitudinal Strain in Patients without Structural Heart Disease
Source: J Clin Med. 2024 Mar 20;13(6):1796. doi: 10.3390/jcm13061796 (PMC10971011; doi:10.3390/jcm13061796)
Supplement: Supplementary file 1 [file jcm-13-01796-s001.zip › jcm-2869668-supplementary.pdf]

Table S1: Baseline Biochemical Characteristics of Patients in The Groups

	GROUP 1<br>N:54	GROUP 2<br>N:33	GROUP 3<br>N:35	P value
**Haemoglobin (mg/dL)**	13.9 ± 1.4	14.2 ± 1.5	14.2 ± 1.6	0.400
**HCT (%)**	40.1 ± 3.8	40.8 ± 3.7	41.4 ± 4.2	0.316
**Leukocytes (/mm^3)**	7424.8 ± 1118.9	7103.0 ± 1689.7	6994.3 ± 1782.5	0.481
**Platelets (x10^3/mm^3)**	255.6 ± 54.1	231.6 ± 59.6	264.5 ± 74.6	0.079
**LDL cholesterol (mg/dl)**	124.4 ± 30.4	127.3 ± 27.4	130.9 ± 38.3	0.641
**HDL cholesterol (mg/dl)**	54.2 ± 10.8	52.6 ± 11.6	50.9 ± 13.4	0.423
**Glucose (mg/dL)**	93 ± 18	91 ± 20	92 ± 22	0.662
**HbA1c (%)**	5.8 ± 0.9	5.8 ± 0.8	5.9 ± 1.1	0.678
**TSH (mIU/L)**	2.4 ± 1.3	2.6 ± 1.9	2.5 ± 1.8	0.924
**fT4 (ng/dl)**	1.1 ± 0.2	1.3 ± 0.6	1.2 ± 0.2	0.742
**BUN (md/dL)**	12.3 ± 3.7	12.4 ± 2.8	12.9 ± 4.1	0.757
**Kreatinine (mg/dL)**	0.8 ± 0.16	0.8 ± 0.1	0.8 ± 0.2	0.491
**Sodium (mmol/L)**	141.7 ± 2.5	142.1 ± 2.4	141.8 ± 2.5	0.865
**Potasium (mmol/L)**	4.6 ± 0.4	4.6 ± 0.4	4.7 ± 0.4	0.221

BUN: blood urea nitrogen, fT4: free thyroxine four, HbA1c: hemoglobin A1c, HCT: hematocrit, HDL: high-density lipoprotein, LDL: low-density lipoprotein, TSH: thyroid stimulating hormone.

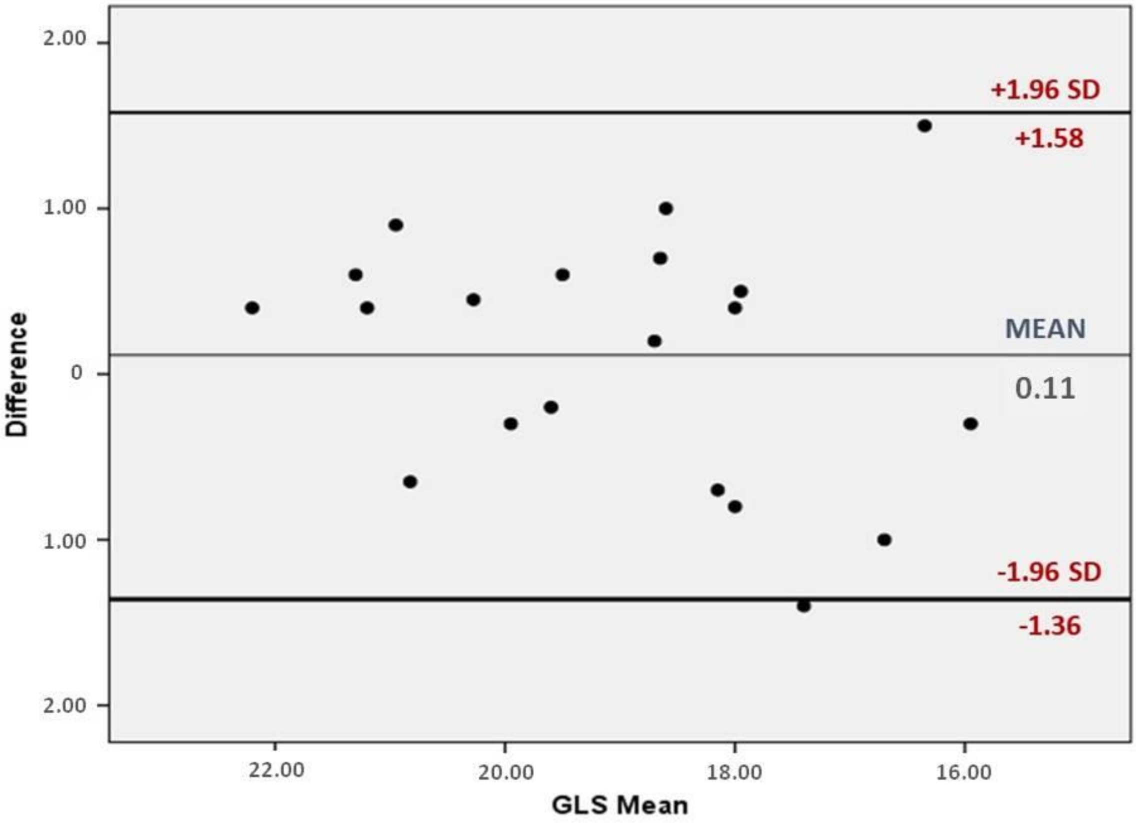

Figure S1. The Blant–Altman Analysis.
